# High Weight Loss during Radiation Treatment Changes the Prognosis in Under-/Normal Weight Nasopharyngeal Carcinoma Patients for the Worse: A Retrospective Analysis of 2433 Cases

**DOI:** 10.1371/journal.pone.0068660

**Published:** 2013-07-15

**Authors:** Lu-Jun Shen, Chen Chen, Bo-Fei Li, Jin Gao, Yun-Fei Xia

**Affiliations:** 1 State Key Laboratory of Oncology in Southern China, Cancer Center, Sun Yat-sen University, Guangzhou, People’s Republic of China; 2 Department of Radiation Oncology, Cancer Center, Sun Yat-sen University, Guangzhou, People’s Republic of China; 3 Zhongshan School of Medicine, Sun Yat-sen University, Guangzhou, People’s Republic of China; Northwestern University Feinberg School of Medicine, United States of America

## Abstract

**Background:**

Although weight loss is common in nasopharyngeal carcinoma (NPC) patients receiving radiotherapy, the prognostic influence of weight loss and its impact modified by body mass index (BMI) are still unclear.

**Methods:**

2433 NPC patients receiving radical radiotherapy at Sun Yat-sen University Cancer Center from November, 2000 to December, 2004 were enrolled. Weight change during radiation treatment was categorized into high weight loss (HWL) and low weight loss (LWL). The associations of HWL with overall survival (OS) and disease-specific survival (DSS) were analyzed by Cox regression.

**Results:**

Among underweight patients, HWL was independently associated with poor OS (hazard ratio [HR], 2.06; 95% CI 1.36–3.11) and DSS (HR, 2.27; 95% CI 1.38–3.73), as compared with LWL, after adjusting for covariates. In normal weight patients, the impact of HWL on OS (HR, 1.47; 95% CI 1.19–1.80) and DSS (HR, 1.59; 95% CI 1.24–2.03) was moderate. Among overweight/obese patients, no significant association between HWL and OS (HR, 1.22; 95% CI 0.95–1.55), or DSS (HR, 1.23; 95% CI 0.93–1.64) was found.

**Conclusion:**

Except for overweight/obese patients, high weight loss during radiation treatment was independently associated with poor survival in NPC. This impact was more prominent in the underweight patient group.

## Introduction

Nasopharyngeal carcinoma (NPC) is an endemic head and neck epithelial malignancy. Approximately 60% patients with nasopharyngeal carcinoma present stage III or IV disease at initial diagnosis [1], [2]. Although the treatment effect of NPC has improved primarily due to the progress in diagnostic imaging, radiation techniques and chemotherapy regimens, 20–30% of the patients will die due to cancer recurrence or/and distant metastasis [3]–[5]. Identification of those high-risk patients may provide new clues in developing clinical intervention to improve their survival.

Body mass index (BMI), a commonly utilized measure for indicating nutritional status in adults, has been shown closely associated with the prognosis of NPC patients [6], [7]. Various studies have focused on the striking association between low BMI at diagnosis and poor prognosis due to higher local-regional recurrence and mortality. As emphasized by Shen et al., the hazard ratio for death was 0.66 for overweight (95% CI, 0.48 to 0.90) and 0.47 (95% CI, 0.23 to 0.97) for obese patients comparing to the baseline of normal weight or underweight patients [7].

In contrast to the considerable amount of research on BMI, the impact of body weight loss on NPC recurrence and death has not been addressed. More than 60% of NPC patients receiving curative-intent radiotherapy suffered from a weight loss greater than 5% during the treatment [8], [9]. Advanced tumors, concurrent chemotherapy, and high BMI are important predisposing factors of weight loss, and it is reported that a critical weight loss (>5%) during the radiation was associated with poorer treatment tolerance and worse prognosis in head and neck cancer (HNC) patients [10], [11].

Is weight loss also an unfavorable prognostic factor for NPC? Since BMI affects both weight loss and survival among NPC patients [9], analysis combining these two weight-related factors may be required. Therefore, in this study, we investigated the prognostic influence of weight loss and its corresponding potential effect-modification by BMI.

## Patients and Methods

### Patients

The medical records of 2820 newly diagnosed NPC patients without distant metastasis in Sun Yat-sen University Cancer Center (SYSUCC) from November, 2000 to December, 2004 were reviewed. Sun Yat-sen University Cancer Center Hospital Ethics Committee approved this study (No. YP201012). This was a retrospective analysis of routine data and therefore we requested and were granted a waiver of individual informed consent from the ethics committee. The data were collected by trained SYSUCC interviewers and analyzed anonymously. All patients received radical radiotherapy and completed the prescribed course of treatment. Exclusion criteria are any of the following: (i) missing weight measurement at baseline and/or at the end of radiotherapy (200); (ii) follow-up period less than 5 years (178); (iii) age less than 18 years old (9). A total of 2433 patients were enrolled. All patients received routinely nasopharyngeal CT or MRI examination before treatment and were staged according to the sixth edition of UICC staging system [12].

### Measurement and Grouping

**Figure 1:**
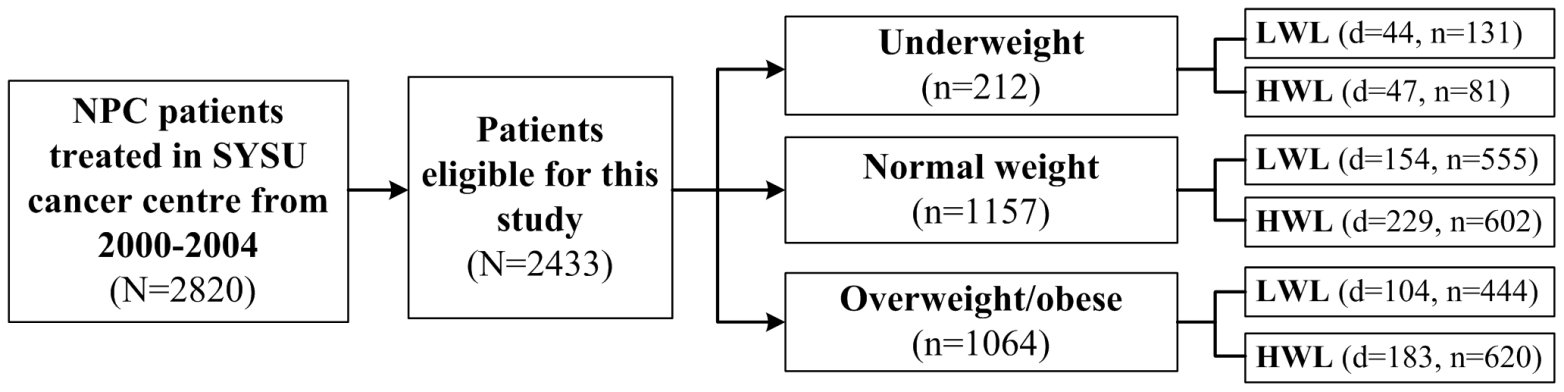
Flowchart of study design. A total of 2433 patients met the enrollment criteria. The population were first divided into 2×3 subgroups by BMI levels and weight loss status, and analyzed in the multiple-adjusted Cox model. Stratified analysis by BMI levels was further conducted to determine the impact of high weight loss during treatment for NPC patients with different BMI levels. Footnote: d, death; n, number of patients.

Pre-radiation treatment (pre-RT) weight was measured within 7 days before radiotherapy (RT), and post-radiation treatment (post-RT) weight was measured within 7 days after completion of RT. BMI was defined as pre-RT weight (kg) divided by the square of height (meter) and was categorized according to the WHO recommendations for Asian population [13]; because the proportion of obese patients was relatively small (6.5%), we merged overweight and obese patients and obtained three BMI groups: <18.5 kg/m^2^, underweight, UW; 18.5–<23.0 kg/m^2^, normal weight, NW; ≥23.0 kg/m^2^, overweight/obese, OW (Figure 1).

Weight loss (ΔW) was calculated as the relative percent of weight changes between weight measurement before and after the radiation treatment,
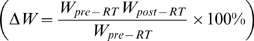



A number of studies suggest that a greater than 5% body weight loss during radiation for HNC patients indicates malnutrition and is clinically meaningful [9], [14], [15]. ΔW was accordingly categorized into high weight loss (HWL; ≥5%) and low weight loss (LWL; <5%).

### Treatment

Radiotherapy included two-dimensional conformal radiotherapy (2D-CRT), three-dimensional conformal radiotherapy (3D-CRT), and intensity-modulated radiotherapy (IMRT). Opposing lateral facial-cervical fields were used in the 2D-CRT to cover the nasopharynx and upper cervical lymphatic drainage region, with 1 lower anterior cervical field to cover the lower cervical region. After 36 to 40 Gy, opposing lateral preauricular fields were used for the primary region, and anterior split neck fields were used for the cervical region. The primary tumor was irradiated to a dose of 60 to 78 Gy. The dose of prophylactic irradiation for patients with 2D-CRT was 50–54 Gy to the prophylactic areas. For 3D-CRT, the total prescribed dose was 66–72 Gy to the gross tumor volume of nasopharynx (GTVnx), 60 to 70 Gy to the region involved by the metastatic lymph nodes (GTVnd), 60 Gy to high risk microscopic (CTV1, the GTVnx and an additional 5- to 10-mm margin), and 50–54 Gy to the low risk microscopic (CTV2). For IMRT, the target definition and delineation were the same as for 3D-CRT. The prescription dose was 68 Gy to the GTVnx, 60 to 64 Gy to the GTVnd of neck, 60 Gy to the CTV1, and 54 Gy to CTV2.

Chemotherapy included induction chemotherapy, concomitant chemotherapy, and adjuvant chemotherapy. The induction or adjuvant chemotherapy regimen was fluorouracil (5-Fu) plus cisplatin. The concurrent chemotherapy regimens were either 5-Fu plus cisplatin or cisplatin alone; the 5-Fu plus cisplatin regimen was 70 to 100 mg/m^2^ of cisplatin on day 1 plus 500 to 750 mg/m^2^ 5-Fu on day 2 till day 5 every 3–4 weeks, for 2 to 3 cycles, and the cisplatin regimen was 30 to 40 mg/m^2^ of cisplatin every week, for 6 to 7 cycles.

### Follow-up and End Points

After completion of RT, patients were followed up every 3 months for the first 3 years by clinic visits, telephone, letter and so on. The intervals gradually increased to 6–12 months after 3 years. The data of follow-up was last reviewed on February 2011.

The primary outcomes were overall survival (OS) and disease-specific survival (DSS). Secondary outcomes were local-regional recurrence free survival (LRFS) and distant metastasis free survival (DMFS). OS was defined as time from diagnosis to death by any causes. DSS was defined as the time from the start of RT to death caused by disease progression or treatment-related complications. LRFS was defined as time from the start of RT to the first occurrence of locoregional failure or death from the primary cancer without a documented site of recurrence or metastasis. DMFS was defined as time from the start of RT to the first occurrence of distant failure.

### Statistical Analysis

Wilcoxon rank sum and Chi-square test were used to compare ordinal and categorical variables between groups, respectively. Rates of DSS, OS, LRFS, and DMFS were estimated by means of the Kaplan-Meier method and were compared between the subgroups with the use of the log-rank test. Cox regression model including the two main effect parameters (weight loss and BMI; in categorical form) and their interaction effect parameters was utilized to test the interaction effect between weight loss status and BMI levels. The multiple-adjusted Cox regression model was used to evaluate the survival of 2×3 weight-based subgroups divided by weight loss status (HWL vs LWL) and BMI levels (UW vs NW vs OW), with the covariates including age, sex, UICC T stage, UICC N stage, and treatment (with or without chemotherapy) [16], [17]; the 2×3 subgroups were as follows: NW+LWL (group 1, reference), NW+HWL (group 2), UW+LWL (group 3), UW+HWL (group 4), OW+LWL (group 5), and OW+HWL (group 6). Stratified multiple-adjusted Cox model by BMI levels was further conducted to evaluate the prognostic significance of weight loss status in each BMI group. A P value <0.05 was considered significant. Statistical analysis was performed using SPSS 20.0 software.

## Results

### Characteristics of the Population

Listed in Table 1 were baseline characteristics of patients. The median age was 46 years (range, 18–78 years). Among all the patients, 2166 (89.0%) had undifferentiated non-keratinizing carcinoma, 203 (8.3%) had differentiated non-keratinizing carcinoma and 64 (2.6%) had other types; 983 (40.4%) had early-stage disease (stage I/II), and 1450 (59.6%) had advanced disease (stage III/IV). All patients received definitive-intent radiotherapy during treatment, with 2194 (90.2%) patients treated with 2D-CRT, 63 (2.6%) patients with 3D-CRT, and 176 (7.2%) patients with IMRT. The duration of RT ranged from 42 to 90 days (median, 50 days). Induction chemotherapy, concurrent chemotherapy and adjuvant chemotherapy was delivered in 830 (34.1%), 504 (20.7%) and 11 (0.5%) patients, respectively. The median duration of follow-up was 85 months (range, 2–125 months).

**Table 1:** Baseline characteristics by weight loss status in patients with nasopharyngeal carcinoma (n = 2433).

Patient characteristics	All		LWL		HWL			P
Duration of RT(days, mean ± SD)	51.0±6.3		50.5±5.9		51.4±6.5			0.072^Ψ^
	No.	%	No.	%	No.	%		
Age, years							0.210	
<46	1285	52.8	613	54.2	672	51.6		
≥46	1148	47.2	518	45.8	630	48.4		
Gender							0.099	
Male	1851	76.1	877	77.6	974	74.8		
Female	582	23.9	253	22.4	329	25.2		
UICC Stage							<0.001	
I	127	5.2	80	7.1	47	3.6		
II	856	35.2	425	37.6	431	33.1		
III	979	40.2	430	38.1	549	42.1		
IV	471	19.4	195	17.3	276	21.2		
UICC T Stage							0.037	
T1	395	16.2	204	18.1	191	14.7		
T2	1023	42.0	485	42.9	538	41.3		
T3	621	25.5	273	24.2	348	26.7		
T4	394	16.2	168	14.9	226	17.3		
UICC N Stage							<0.001	
N0	635	26.1	354	31.3	281	21.6		
N1	976	40.1	439	38.8	537	41.2		
N2	732	30.1	303	26.8	429	32.9		
N3	90	3.7	34	3.0	56	4.3		
BMI							<0.001	
Underweight	212	8.7	131	11.6	81	6.2		
Normal weight	1157	47.6	555	49.1	602	46.2		
Overweight/obese	1064	43.7	444	39.3	620	47.6		
Treatment							<0.001	
RT	1088	44.7	579	51.2	509	39.1		
RT+CT	1345	55.3	551	48.8	794	60.9		

Footnote: UICC, International Union Against Cancer; BMI, body mass index; RT, radiotherapy; CT, chemotherapy; LWL, low weight loss; HWL, high weight loss; Ψ, result of t-test; P<0.05 was considered statistically significant.

For the entire population, the mean BMI was 22.60 kg/m^2^. A total of 212 patients (8.7%) were underweight, 1157 (47.6%) were normal weight, and 1064 (43.7%) were overweight/obese. During radiation treatment, the mean weight loss was 4.33 kg, and 53.6% (1303) patients had high weight loss (HWL; ΔW≥5%). The proportion of HWL was higher in patients with advanced T stage, N stage, high BMI level and who received chemotherapy. No significant differences in age and sex were found across the weight loss subgroups (Table 1).

### BMI, Weight Loss, and Survival

572 (23.5%) of the 2433 patients experienced cancer recurrence, 166 (6.8%) had distant metastasis, and 761 (31.3%) died. The 1-, 3-, and 5-year overall survival (OS) rates for the whole population were 96.8%, 83.8%, and 74.6%, respectively.

In unadjusted analysis, underweight patients had significantly lower 5-year OS rate compared to normal weight (65% vs 73%, P = 0.004) and overweight/obese patients (65% vs 78%, P<0.001); HWL patients presented with significantly lower 5-year OS rate compared to LWL patients (P<0.001). Age, sex, UICC T stage, UICC N stage, and treatment also significantly influenced OS (P<0.001 for all). Results on 5-year DSS and LRFS were similar to that of OS, while no difference in DMFS was found across the weight loss groups (Table 2).

**Table 2:** Unadjusted analysis for 5-year OS, DSS, LRFS, and DMFS rates.

	5-yr OS (%)	*P*	5-yr DSS (%)	*P*	5-yr LRFS (%)	*P*	5-yr DMFS (%)	*P*
Age, years								
<46	80	<0.001	80	<0.001	85	<0.001	94	0.324
≥46	69		71		76		94	
Sex								
Male	73	<0.001	75	0.001	78	<0.001	94	0.350
Female	81		81		85		95	
UICC T Stage								
T1–T2	78	<0.001	79	<0.001	82	<0.001	94	0.142
T3–T4	70		72		76		93	
UICC N Stage								
N0–N1	78	<0.001	80	<0.001	82	<0.001	95	0.001
N2–N3	67		69		75		91	
Treatment								
RT	79	<0.001	81	<0.001	82	0.001	97	<0.001
RT+CT	71		72		78		91	
BMI levels								
Underweight	65		67		76		90	
Normal weight	73	<0.001†	75	<0.001†	79	0.004†	94	0.036†
Overweight/obese	78		79		83		95	
Weight loss								
LWL	78	<0.001	81	<0.001	84	<0.001	94	0.891
HWL	71		73		78		94	

Footnote: OS, overall survival; DSS, disease-specific survival; LRFS, local-regional recurrent free survival; DMFS, distant metastasis free survival. Other abbreviation as in Table 1;

† result of joint test.

**Figure 2:**
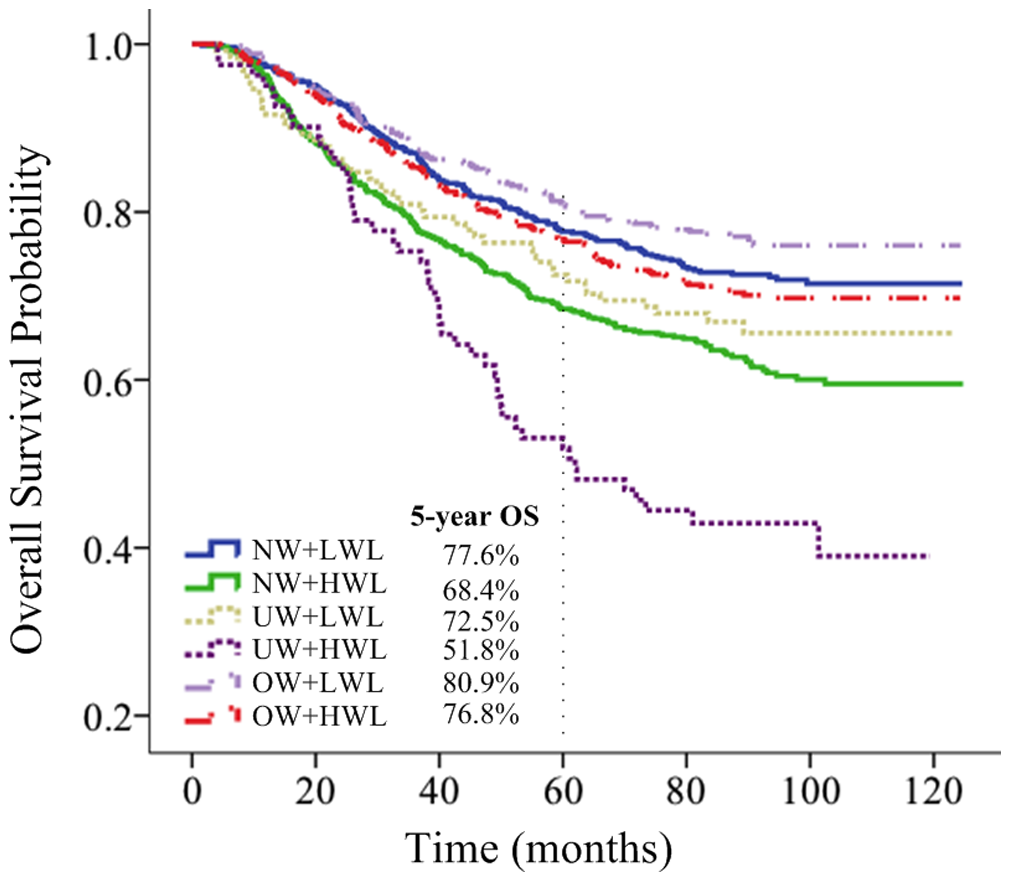
OS for NPC patients after radical radiotherapy in 2×3 grouping by BMI levels and weight loss status.

The BMI×Weight loss interaction terms were tested for OS, and a borderline significance was identified for the differential impact of weight loss between underweight and overweight/obese patients (normal weight×HWL vs underweight×LWL, P = 0.239; overweight/obese×HWL vs underweight×LWL, P = 0.054; Table S1). Therefore, we firstly divided the whole population into 2×3 weight-based subgroups according to BMI and weight loss. OS, DSS and LRFS were identified as significant in unadjusted analysis among the six subgroups, with joint P value <0.001 for all (Figure 2; Figure S1–S2); yet no significant difference was found for DMFS (P = 0.089).

Multiple-adjusted analysis including 2×3 weight-based subgroups, age, sex, UICC T stage, UICC N stage and treatment was performed. Comparing to NW+LWL patients, patients in UW+HWL group had significantly lower OS (HR, 2.56; 95% CI 1.65–3.56), DSS (HR, 2.66; 95% CI 1.82–3.88) and LRFS (HR, 2.62; 95% CI 1.81–3.78); NW+HWL patients had mildly unfavorable OS (HR, 1.45; 95% CI 1.18–1.78), DSS (HR, 1.57; 95% CI 1.22–2.01) and LRFS (HR, 1.44; 95% CI 1.14–1.82). By contrast, UW+LWL, OW+LWL, and OW+HWL patients did not have significantly different OS, DSS, or LRFS in comparison with NW+LWL patients (Table 3).

**Table 3:** Multiple-adjusted analysis for OS and DSS among the whole population (n = 2433).

Variables	OS			DSS		
	HR	95% CI	P	HR	95% CI	P
Age, years			<0.001			<0.001
<46	Ref			Ref		
≥46	1.83	1.58–2.12		1.65	1.40–1.96	
Sex			<0.001			<0.001
Male	Ref			Ref		
Female	0.68	0.57–0.82		0.69	0.56–0.86	
UICC T Stage			<0.001			<0.001
T1–T2	Ref			Ref		
T3–T4	1.35	1.17–1.57		1.34	1.13–1.59	
UICC N Stage			<0.001			<0.001
N0–N1	Ref			Ref		
N2–N3	1.44	1.23–1.67		1.45	1.21–1.74	
Treatment			0.049			0.064
RT	Ref			Ref		
RT+CT	1.18	1.00–1.38		1.23	1.02–1.49	
BMI+Weight loss(2×3 groups)			<0.001†			<0.001†
NW+LWL	Ref			Ref		
NW+HWL	1.45	1.18–1.78	<0.001	1.57	1.22–2.01	<0.001
UW+LWL	1.25	0.89–1.75	0.191	1.23	0.80–1.88	0.348
UW+HWL	2.56	1.65–3.56	<0.001	2.66	1.82–3.88	<0.001
OW+LWL	0.82	0.64–1.05	0.116	0.90	0.67–1.22	0.501
OW+HWL	0.98	0.79–1.22	0.881	1.08	0.83–1.40	0.577

Footnote: Ref, reference; HR, hazard ratio.

† result of joint test.

**Figure 3:**
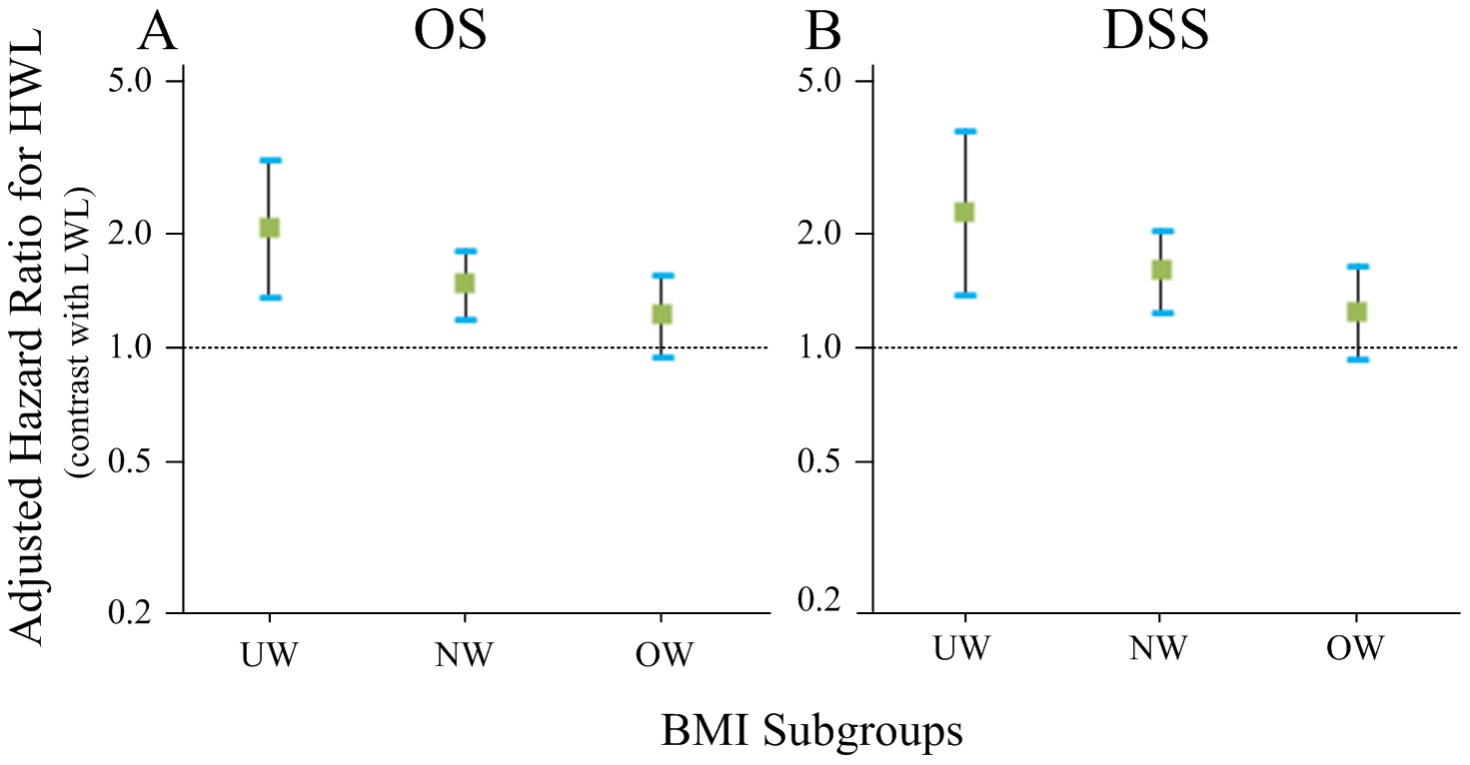
Adjusted hazard ratios of high weight loss for survival in patients stratified by BMI levels. Hazard ratios of high weight loss for overall survival (OS) and disease-specific survival (DSS) in NPC patients receiving radical radiotherapy, stratified by BMI levels. HR was adjusted for age, sex, UICC T stage, UICC N stage, and treatment; 95% CIs were shown.

To assess the prognostic impact of weight loss for patients with different BMI levels, further stratified analysis was conducted (Table 4). Multiple-adjusted analysis showed HWL was independently associated with adverse OS (HR, 2.06; 95% CI 1.36–3.11), DSS (HR, 2.27; 95% CI 1.38–3.73), and LRFS (HR, 2.93; 95% CI 1.75–4.90) among underweight patients. In the subgroup of normal weight patients, HWL was also significant on OS (HR, 1.47; 95% CI 1.19–1.80), DSS (HR, 1.59; 95% CI 1.24–2.03), and LRFS (HR, 1.45; 95% CI 1.15–1.84). Among overweight/obese patients, no significant association between HWL and survival was found (Figure 3).

**Table 4:** Outcomes according to weight loss stratified by BMI levels in multiple-adjusted Cox model adjusted for age, sex, UICC T stage, UICC N stage, and treatment.

Weight Change	No.	OS		DSS		LRFS		DMFS	
		HR (95% CI)	P	HR (95% CI)	P	HR (95% CI)	P	HR (95% CI)	P
Underweight (n = 212)									
LWL	131	Ref		Ref		Ref		Ref	
HWL	81	2.06 (1.36–3.11)	0.001	2.27 (1.38–3.73)	0.001	2.93 (1.75–4.90)	<0.001	0.60 (0.23–1.53)	0.284
Normal weight(n = 1157)									
LWL	555	Ref		Ref		Ref		Ref	
HWL	602	1.47 (1.19–1.80)	<0.001	1.59 (1.24–2.03)	<0.001	1.45 (1.15–1.84)	0.002	1.15 (0.74–1.78)	0.537
Overweight/obese (n = 1064)									
LWL	444	Ref		Ref		Ref		Ref	
HWL	620	1.22 (0.95–1.55)	0.117	1.23 (0.93–1.64)	0.153	1.23 (0.93–1.63)	0.146	0.92 (0.55–1.54)	0.761

Footnote: Ref, reference; HR, hazard ratio. P<0.05 was considered statistically significant.

## Discussion

BMI is one of the criteria commonly used in the assessment of nutritional status, whose prognostic impact has been extensively studied among NPC patients [6], [7]. However, percentage weight loss, a sensitive and specific tool in assessment of recently developed malnutrition [18], [19], has been shown correlated with poor prognosis in head and neck cancer patients [11], [20], and should be taken into account in NPC prognosis.

Currently, no data specifically focused on the prognostic impact of weight loss among NPC patients was available. In our series, it’s intriguing to find a potential differential impact of weight loss between underweight and overweight/obese patients (P = 0.054). This indicates that a 2×3 grouping by BMI levels and weight loss and further stratified analysis by BMI levels are necessary to determine the impact of weight loss in NPC. Using the 2×3 grouping, we found that UW+HWL group had significantly lower OS comparing to the other five subgroups. Further stratified analysis by BMI levels showed HWL was independently associated with unfavorable OS and DSS among underweight patients (HR, 2.06; 95% CI 1.36–3.11; HR, 2.27; 95% CI 1.38–3.73). In normal weight patients, the impact of HWL was moderate (HR, 1.47; 95% CI 1.19–1.80; HR, 1.59;95% CI 1.24–2.03); among overweight/obese patients, such impact was weak (HR, 1.22; 95% CI 0.95–1.55; HR, 1.23; 95% CI 0.93–1.64). These novel observations underscore the importance of pretreatment BMI status in the interpretation of weight loss and, to our knowledge, represent the first data showing that high weight loss adversely impacts on survival in NPC patients.

The reason underlying the differential prognostic impact of weight loss on NPC patients of different BMI levels may mainly involve malnutrition. During the radiation treatment, underweight patients were more susceptible to severe side effects, such as mucositis, dysphagia, etc., than normal weight patients, which may affect their food intake and lead to body wasting [21]–[23]. Further high weight loss during the treatment may serve as a prominent indicator of malnutrition [20], which is known to be correlated with compromised immunity [23], [24], unplanned treatment breaks and poor treatment outcomes in head and neck cancer [25]–[27]; by contrast, low weight loss in underweight patients may represent a well-tolerant nutritional status and is therefore associated with better prognosis [7], [28]. The unfavorable impact of weight loss was moderate in normal weight patients, and even weaker in overweight/obese patients; some studies reported that NPC patients with normal or higher baseline BMI may be more resistance to malnutrition [7], [29], [30], and this can partly explain our results. Apart from the impact of treatment, factors like advanced tumors, severe comorbidities and even insomnia can also contribute to body weight loss of NPC patients during radiation treatment [9]. Patients with advanced tumor are more likely to develop cancer cachexia syndrome, which is characterized by nutritionally irreversible weight loss and will greatly increase the mortality rate [31]. Comorbidities like diabetes, chronic obstructive pulmonary disease, and cardiovascular diseases will also worsen the prognosis of NPC patients and, on the other hand, exacerbate body wasting [32], [33]. A recent study of 1001 newly diagnosed NPC patients showed that 15.5% elderly NPC patients (≥70 years) presented with moderate to severe comorbidities (ACE-27 score≥2) before radiation treatment; these patients had lower OS (HR, 2.63, 95% CI 1.45–4.76) than patients had none or mild comorbidities [33]. However, in our population, no patients presented with cachexia before treatment, and the proportion of elderly patients was rather small (16pts, 0.8%). Therefore, this explanation may not be the main consideration.

Upon 2×3 grouping based on assortment of BMI and weight loss, we found UW+HWL patient group had lowest OS and DSS as compared with the other five groups, while e.g. the difference in survival rate between UW+LWL and NW+LWL patients was small (Figure 2). These data suggested that appropriate intervention towards weight loss, or malnutrition might be beneficial to underweight patients. For the past decade, there has been ample evidence that adequate nutrition support (NS) before and during radiotherapy can decrease the impact of side effect of treatment, minimize weight loss, and improve outcome for head and neck cancer patients [34], [35]. Weight loss of more than 1–2% per week, or 5% in less than a month, should prompt further patient assessment, nutritional counseling, and more aggressive interventions [36]. However, Rabinovitch et al. analyzed the data of 1073 advanced HNC patients treated with definitive radiotherapy and found that patients with nutrition support during treatment had better 5-year locoregional control and 5-year overall survival rate as compared with patients receiving pre-treatment nutrition support, and that the pre-treatment nutritional support group even had poorer survival as compared with those with no nutritional support at all [37]. Therefore, how and when to adopt these nutritional interventions in NPC await further study.

Our study has several limitations. First, it is a retrospective study. Second, the proportion of obese patients in this study was relatively small (6.5%), which might impede our interpretation of the prognostic value of HWL in this group; therefore, we merged the overweight and obese group. Third, the modes of chemotherapy applied varied, which might have a confounding effect. For these reasons, we need to validate our finding in a multi-institutional prospective study in the future.

### Conclusion

Underweight NPC patients with high weight loss during radiation had worst survival compared with other subgroups assorted by BMI levels and weight loss. High weight loss is independently associated with increased mortality among underweight and normal weight, but not overweight/obese, NPC patients.

## Supporting Information

Figure S1 **DSS for NPC patients after radical radiotherapy in 2×3 grouping by BMI levels and weight loss status.**
(TIF)

Figure S2 **LRFS for NPC patients after radical radiotherapy in 2×3 grouping by BMI levels and weight loss status.**
(TIF)

Table S1 **Test for interaction between weight loss and BMI levels, T stage, N stage, age and treatment on OS.**
(DOCX)
