# Correction: High Weight Loss during Radiation Treatment Changes the Prognosis in Under-/Normal Weight Nasopharyngeal Carcinoma Patients for the Worse: A Retrospective Analysis of 2433 Cases

**DOI:** 10.1371/annotation/3dc37158-1f60-436e-9bd5-2a822aa2c9cb

**Published:** 2013-12-16

**Authors:** Lu-Jun Shen, Chen Chen, Bo-Fei Li, Jin Gao, Yun-Fei Xia

In the Patients and Methods section, there was an error in the first equation. A minus sign was omitted between pre-RT and W. Please view the complete, correction equation here: http://www.plosone.org/corrections/pone.0068660.001.cn.tif

