# Supplementary figures and images for: High Weight Loss during Radiation Treatment Changes the Prognosis in Under-/Normal Weight Nasopharyngeal Carcinoma Patients for the Worse: A Retrospective Analysis of 2433 Cases
Source: PLoS One. 2013 Jul 15;8(7):e68660. doi: 10.1371/journal.pone.0068660 (PMC3711826; doi:10.1371/journal.pone.0068660)

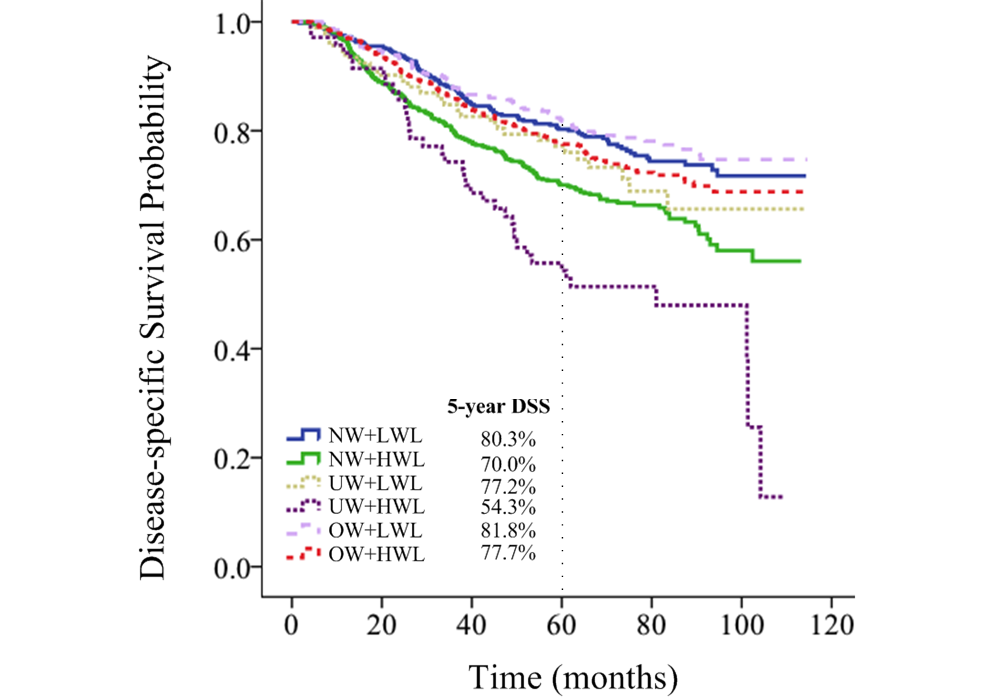

Supplement: Figure S1 — DSS for NPC patients after radical radiotherapy in 2×3 grouping by BMI levels and weight loss status. (TIF) [file pone.0068660.s001.tif]

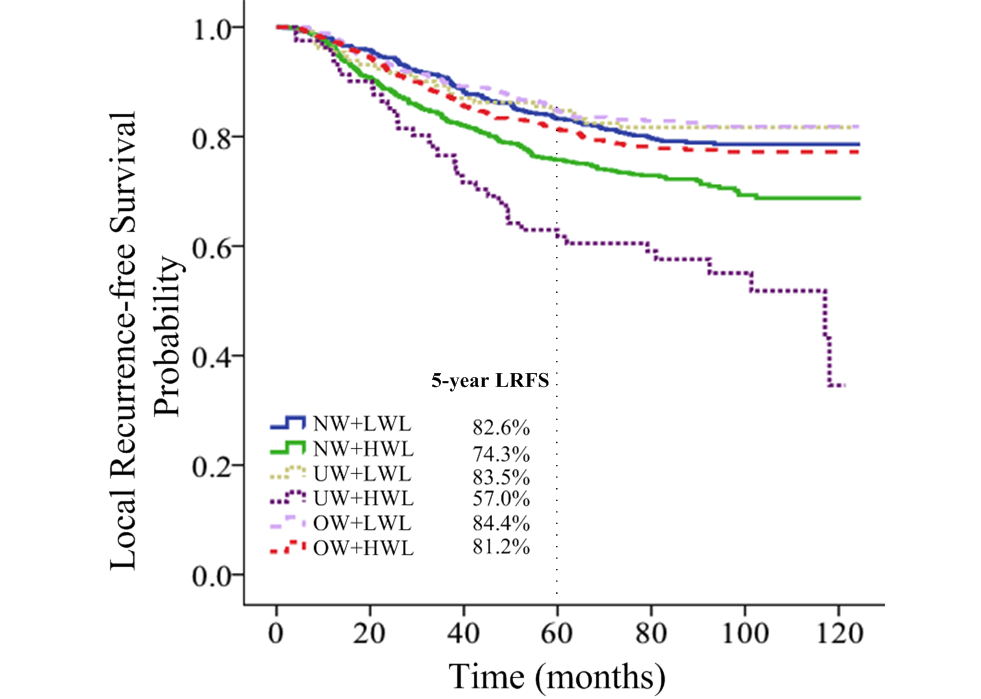

Supplement: Figure S2 — LRFS for NPC patients after radical radiotherapy in 2×3 grouping by BMI levels and weight loss status. (TIF) [file pone.0068660.s002.tif]
