# Supplementary material for: High Weight Loss during Radiation Treatment Changes the Prognosis in Under-/Normal Weight Nasopharyngeal Carcinoma Patients for the Worse: A Retrospective Analysis of 2433 Cases
Source: PLoS One. 2013 Jul 15;8(7):e68660. doi: 10.1371/journal.pone.0068660 (PMC3711826; doi:10.1371/journal.pone.0068660)
Supplement: Table S1 — Test for interaction between weight loss and BMI levels, T stage, N stage, age and treatment on OS. (DOCX) [file pone.0068660.s003.docx]

Table S1. Test for interaction between weight loss and BMI levels, UICC T stage, UICC N stage, age and treatment on OS

| Test for interaction and variable | HR | 95%CI | P |
| --- | --- | --- | --- |
| **BMI levels and Weight loss** |  |  |  |
| BMI levels |  |  |  |
| Normal weight vs Underweight | 0.767 | 0.549-1.073 | 0.121 |
| Overweight/obese vs Underweight | 0.635 | 0.446-0.903 | 0.012 |
| Weight loss |  |  |  |
| HWL vs LWL | 1.50 | 1.318-3.001 | 0.001 |
| BMI×Weight loss |  |  |  |
| Normal weight×HWL vs Underweight×LWL | 0.759 | 0.480-1.201 | 0.239 |
| Overweight/obese×HWL vs Underweight×LWL | 0.661 | 0.434-1.006 | 0.054 |
| **UICC T stage and Weight loss** |  |  |  |
| T3-T4 vs T1-T2 | 1.579 | 1.260-1.979 | <0.001 |
| HWL vs LWL | 1.454 | 1.186-1.782 | <0.001 |
| T stage×WL | 0.905 | 0.676-1.210 | 0.499 |
| **UICC N stage and Weight loss** |  |  |  |
| N2-N3 vs N0-N1 | 1.385 | 1.148-1.672 | 0.001 |
| HWL vs LWL | 1.543 | 1.222-1.948 | <0.001 |
| N stage×WL | 0.971 | 0.722-1.307 | 0.848 |
| **Age and Weight loss** |  |  |  |
| ≥46 vs <46 | 1.469 | 1.172-1.843 | 0.001 |
| HWL vs LWL | 1.829 | 1.455-2.300 | <0.001 |
| Age×WL | 0.908 | 0.676-1.220 | 0.522 |
| **Treatment and Weight loss** |  |  |  |
| RT+CT vs RT alone | 1.730 | 1.306-2.292 | <0.001 |
| HWL vs LWL | 1.646 | 1.252-2.163 | <0.001 |
| Treatment×WL | 0.751 | 0.527-1.069 | 0.112 |

Footnote: BMI was categorized in to three groups: (<18.5 kg/m^2^, underweight; 18.5-< 23.0 kg/m^2^, normal weight; >23.0 kg/m^2^, overweight & obesity). Cox regression model including the two main effect parameters (in categorical form) and their interaction effect parameter(s) was utilized to test the interaction effect between weight loss status and BMI levels, UICC T stage, UICC N stage, age and treatment. WL, weight loss, LWL, low weight loss; HWL, high weight loss; RT, radiotherapy; CT, chemotherapy.
